# Effects of intermittent fasting on markers of body composition and mood state

**DOI:** 10.1186/1550-2783-11-S1-P25

**Published:** 2014-12-01

**Authors:** Sara Hayward, Jordan Outlaw, Stacie Urbina, Bailey Burks, Josh Holt, Matt Stone, Alena Regeski, Maegon Saur, Jennifer Ander, Lem Taylor, Cliffa Foster, Colin Wilborn

**Affiliations:** 1 University of Mary Hardin-Baylor, Belton, Texas, USA

## Background

Optimal athletic performance is often linked to body composition, therefore depending on sport and position, a decrease in body fat would lean toward an increase in athletic fitness. A recent trend in decreasing body fat while maintaining muscle has emerged, called intermittent fasting. Some research has suggested that ingesting calories only in a certain time frame during the day (8 hour, 10hour window) would put the body into fat metabolism while avoiding muscle catabolism during the remaining fasting hours. The purpose of this study is to investigate the effects of intermittent fasting and resistance training on body composition, mood state, and resting energy expenditure.

## Methods

Twenty-four participants (males n= 8 and females n= 16) volunteered to participate in this study and were randomly assigned to one of three groups: Resistance training only (RT), where they performed 4 upper and lower body split workouts a week, Intermittent Fasting only (IMF), this group consumed calories in an 8 hour window while maintaining normal free living activity, and Intermittent Fasting plus Resistance Training (IMFRT), this group participated in 4 workouts per week as well as only ingesting calories within the 8 hour window. Participants completed baseline testing consisting of Resting Energy Expenditure on a TrueOne ParvoMedics metabolic cart to determine caloric need, as well as measuring body composition using dual energy x-ray absorptiometry scan (DEXA). For the next 30 days the participants followed their group protocol, while coming in at day 15 for a weigh-in as well as a completion of a mood state questionnaire. The final testing session occurred at the end of the 30-day workout and/or dietary intervention. Data analysis was performed using a MANCOVA [3(group)x 2(time) gender as a covariate], as well as Independent Samples T-Test to determine individual group differences, post hoc test were set at p<0.05. Consent to publish the results was obtained from all participants.

## Results

There was a significant linear interaction shown for lean mass from Day 1 to Day 30 (p=0.002). A significant difference in Resting Energy Expenditure was also found for the groups (p=.010). Independent samples t-tests found a significant difference in weight (p=0.037) as well as fat mass (p=0.036) between the Intermittent Fasting plus Resistance Training group compared to the Resistance training only group. There were no significant differences shown between the Intermittent Fasting plus Resistance Training and Intermittent Fasting only. Also when compared, the Intermittent Fasting and Resistance Training only group had no significant differences. A Nonparametric tests reviled no significant differences between mood states for the groups.

## Conclusion

An 8-hour eating and 16-hour fasting day resulted in a decrease in fat mass as well as weight for the Intermittent Fasting plus Resistance Training group when compared to the Resistance Training only group. On the other hand no differences were found between the Resistance Training only group and Intermittent Fasting group, hinting to that intermittent fasting alone may not be affective in decreasing body fat percent. However, when paired with resistance training, lean mass can be retained and/or enhanced while decreasing body fat, thus enhancing body composition.

